# A Fatal Case of Meningitis Due to Infection of the Sphenoidal Sinus by Pfeiffer's Bacillus, with a Short Review of Methods of Treatment

**Published:** 1936

**Authors:** C. Elaine Field, Herbert Rogers

**Affiliations:** Assistant Medical Officer, North Middlesex County Hospital; Assistant Pathologist to the Hospital


### A FATAL CASE OF MENINGITIS DUE TO INFECTION OF THE SPHENOIDAL SINUS BY PFEIFFER'S BACILLUS, WITH A SHORT REVIEW OF METHODS OF TREATMENT.

BY

C. Elaine Field, M.B., B.S.,
*Assistant Medical Officer, North Middlesex County Hospital,*

AND

Herbert Rogers, M.D.,
*Assistant Pathologist to the Hospital.*

---

Cases of meningitis attributed to infection by organisms resembling Pfeiffer's Bacillus are not uncommon, and over 300 have been reported. The mortality is given by Bloom[1] as 92 per cent., but 46 cases of recovery are on record.

Meningitis due to Pfeiffer's Bacillus is usually stated to be a " primary " infection of the meninges, but the experimental work of Blake and Cecil on the production of broncho-pneumonia by this organism makes it appear probable that sinus infection must be very common.

The occurrence of this form of meningitis, however, is sporadic, and only rarely associated with epidemic influenza, which is now regarded as being due to a filter-passing organism.

This sporadic incidence of the disease has resulted in treatment remaining empirical or a matter of desperation.

The recorded cases of recovery during the last ten years, and the methods of treatment, are as follows :—

| *Author.* | *Year.* | *Age.* | *Sex.* | *Treatment.* |
|---|---|---|---|---|
| Turner. *Jour. Iowa Med. Soc.*, 16, 490. | 1926 | 6½ years. | F. | Lumbar punctures, antimeningococcal serum. |
| Signorelli. *Proc. Louisiana State Pediat. Soc.* | 1929 | 7 years. | M. | Lumbar punctures, antimeningococcal serum. |
| Bloom. *New Orleans Med. Jour.*, 83, 455. | 1931 | 10 months. | F. | Lumbar punctures, antimeningococcal serum. |
| Dabney. *Laryngoscope*, 41, 14. | 1931 | 15 years. | M. | Lumbar punctures, methenamine. |
| Gibbens. *Lancet*, 1, 291. | 1931 | 5 years. | F. | Lumbar punctures, antimeningococcal serum. |
| Mastner and Davidson. *Jour. Michigan Med. Soc.*, 29, 311. | 1931 | 2 months. | F. | Spinal block, laminectomy. |
| Johnson. *Jour. Med. Soc., New Jersey*, 29, 311. | 1932 | 2 years. | F. | Lumbar punctures. |
| Hart. *Canadian Med. Assoc. Jour.*, 27, 163 | 1932 | 3 years. | F. | Lumbar punctures. |
| Hart (second case). *Canadian Med. Assoc. Jour.*, 27, 163. | 1932 | 5½ years. | F. | Lumbar punctures, convalescent serum. |
| Wood. *Brit. Med. Jour.*, 101, July. | 1932 | 6 years. | F. | Lumbar punctures, antimeningococcal serum. |
| Needles. *Jour. Amer. Med. Assoc.*, 99, 1342. | 1932 | 29 years. | M. | Lumbar punctures, antimeningococcal serum. |
| Ward and Wright. *Jour. Exp. Med.*, 55, 233. | 1932 | 2½ years. | M. | Lumbar punctures, anti-influenzal serum. |
| Applebaum. *Jour. Amer. Med. Assoc.*, 102, 513. | 1933 | 14 years. | M. | Lumbar punctures, antimeningococcal serum, anti-influenzal serum. |

| *Author.* | *Year.* | *Age.* | *Sex.* | *Treatment.* |
|---|---|---|---|---|
| Applebaum. *Jour. Amer. Med. Assoc.*, 102, 513. | 1933 | 28 years. | M. | Lumbar punctures, antimeningococcal serum, anti-influenzal serum. |
| Applebaum. *Jour. Amer. Med. Assoc.*, 102, 513. | 1933 | 38 years. | M. | Lumbar punctures, antimeningococcal serum, anti-influenzal serum. |
| Pittman. *Jour. Exp. Med.*, 58, 683. | 1933 | 2 years. | F. | Intraspinal specific Type B serum. |
| Robertson. *St. Bart's. Hosp. Jour.*, 40, 10. | 1933 | 2½ years. | F. | Lumbar punctures. |
| Rittenberg. *Jour. Amer. Med. Assoc.*, 102, 1674. | 1934 | 8 years. | F. | Lumbar punctures, anti-influenzal serum. |
| Duncan and Webb. *Jour. Pediat.*, 12, 216. | 1934 | 8 years. | M. | Lumbar punctures, antimeningococcal serum. |
| Dyke and Little. *Lancet*, 6, 1392. | 1934 | 22 years. | M. | Lumbar punctures. |
| Applebaum. *New York State Med. Jour.*, 35, 215. | 1935 | 47 years. | M. | Intrathecal antimeningococcal serum. |
| Faust and Stein. *Jour. Pediat.*, 52, 743. | 1935 | 11 months. | M. | Forced spinal drainage. |
| White. *Canadian Med. Assoc. Jour.*, 34, 163. | 1936 | 3 years. | F. | Intraspinal meningococcal and anti-influenza sera. |

This list is compiled from the references given by Neal, Jackson, and Applebaum,[2] Faust and Stein,[3] and an additional recent case report.

Repeated lumbar puncture alone or combined with antimeningococcal serum intrathecally and intravenously appears to be the usual method of treatment.

Anti-influenzal serum, an anti-bacterial serum obtained from horses immunized with suspensions of living Pfeiffer's Bacillus isolated from fatal cases of "influenzal" meningitis, is now available in America, and has been used in two cases of recovery reported recently.

In both cases treatment was commenced with antimeningococcal serum.

It is not possible to ascertain the number of fatal cases in which anti-influenzal serum has been employed.

Rittenberg's case[4] (1934), after treatment with antimeningococcal serum during the first two days, received 15 c.c. anti-influenzal serum intrathecally every 8 hours for two days, then every 12 hours for five days, and subsequently once daily.

A total of 750 c.c. of cerebrospinal fluid was removed during treatment and replaced by 565 c.c. of serum.

The treatment in White's[5] case (1936) is summarized as follows: 170 c.c. antimeningococcal serum intraspinally, 40 c.c. intravenously, and 100 c.c. intramuscularly during the first five days without any clinical evidence of improvement being noted.

Anti-influenzal serum was then employed, 30 c.c. being given intraspinally on two successive days. Improvement is said to have been immediate. The "forced spinal drainage" method used by Faust and Stein (1935) is remarkable in that during the first 12 hours 1,325 c.c. of hypotonic saline were given intravenously to an infant of 11 months, yielding 125 c.c. of turbid cerebrospinal fluid by continuous drainage of the theca. The cell count fell from 1,300 cells per c.mm. to 75 cells per c.mm. Following this, the saline was given subcutaneously for some days, and again intravenously in large quantity when an exacerbation of symptoms occurred. The final outcome was a complete recovery.

The following fatal case of Pfeiffer's meningitis, coming under the observation of one of us (C.E.F.), may perhaps emphasize the lack of a readily available specific serum. The probable source of infection in the sphenoidal sinus suggested clinically and observed post-mortem is a feature :—

### Case Report.

A female child, aged 3 years and 10 months, was admitted to the Hospital after an illness of three days characterized by sore throat, severe headache, and vomiting.

The patient was lethargic and delirious ; head retraction, a positive Kernig's sign, and extensor plantar responses were noted.

Examination of the chest and abdomen did not reveal any evidence of disease. The ears showed no abnormality and there was no rash.

Lumbar puncture showed a cloudy fluid not under increased pressure, and 5 c.c. of antimeningococcal serum were given intrathecally.

Two days later lumbar puncture showed increased pressure, and during the following ten days 160 c.c. of cerebro-spinal fluid were withdrawn and replaced by 50 c.c. of normal horse serum.

On the third day of treatment a persistent internal squint developed, but plantar responses became flexor.

Head retraction and Kernig's sign remained positive throughout, and dehydration became marked.

Signs of broncho-pneumonia appeared on the 17th day of the illness, and she died suddenly two days later.

### Laboratory Investigations.

Specimens of cerebro-spinal fluid were examined on six occasions, and in each numerous pus cells, increasing amounts of protein and globulin, with chlorides decreasing to 650 mgrms. per cent. were found.

All specimens contained large numbers of small pleomorphic gram-negative bacilli, many being intracellular.

A scanty growth of small dew-drop colonies appeared after twenty-four hours' incubation on fresh blood and "chocolate" agar plates, becoming fairly profuse after a further twenty-four hours' incubation.

No growth was obtainable on plain agar, but hydrocele fluid added to the blood agar appeared to accelerate the rate of growth.

The organisms did not ferment any of the usual laboratory sugars. Suspensions of the organisms injected intravenously into rabbits produced no untoward effects, and no agglutinins were demonstrable in their sera. The organism can therefore be regarded as Pfeiffer's Bacillus on the morphological and cultural characteristics.

### POST-MORTEM REPORT.

*Brain.*—A thick purulent exudate covered the structures beneath the tentorium and extended in smaller amounts along the sulci on to both cerebral hemispheres.

Similar exudate in small quantity was present around the choroid plexuses of the lateral ventricles.

Smears and cultures contained the same organism isolated from the cerebro-spinal fluid during life which corresponds with Pfeiffer's Bacillus.

*Cranial Sinuses.*—The frontal sinuses and middle ears were found to be clean. The sphenoidal sinus contained thin pus from which a growth of Pfeiffer's Bacillus was obtained.

*Lungs.*—Both lower lobes presented broncho-pneumonic consolidation in their more peripheral zones.

*Other Organs* showed degenerative changes only.

### CONCLUSIONS.

It is suggested that this is a case of meningitis caused by Pfeiffer's Bacillus, and originating as an infection of the sphenoidal sinus.

The desirability of a more detailed study of such cases is urged with a view to ascertaining the value of a more specific treatment and its availability.

We are indebted to Mr. T. H. C. Benians for the animal inoculations, and to Mr. Ivor Lewis, Medical Superintendent of our Hospital, for permission to publish these notes.

### REFERENCES.

[1] Chas. Bloom, " Influenzal Meningitis Amenable to Treatment," *New Orleans Med. and Surg. Jour.*, 33, 445, 1930–31.

[2] Neal, Jackson and Applebaum, *Jour. Amer. Med. Assoc.*, 102, 513, 17th February, 1934.

[3] Faust and Stein, *Arch. Pediat.*, 52, 743, 1935.

[4] Rittenberg, *Jour. Amer. Med. Assoc.*, 102, 1674, 1934.

[5] White, *Canadian Med. Assoc. Jour.*, xxxiv, 163, 1936.

